# Kin recognition in an annual plant

**DOI:** 10.1098/rsbl.2007.0232

**Published:** 2007-06-13

**Authors:** Susan A Dudley, Amanda L File

**Affiliations:** Department of Biology, McMaster University1280 Main Street West, Hamilton, Ontario, Canada L8S 4K1

**Keywords:** phenotypic plasticity, root allocation, kin selection, competition, plant, kin recognition

## Abstract

Kin recognition is important in animal social systems. However, though plants often compete with kin, there has been as yet no direct evidence that plants recognize kin in competitive interactions. Here we show in the annual plant *Cakile edentula*, allocation to roots increased when groups of strangers shared a common pot, but not when groups of siblings shared a pot. Our results demonstrate that plants can discriminate kin in competitive interactions and indicate that the root interactions may provide the cue for kin recognition. Because greater root allocation is argued to increase below-ground competitive ability, the results are consistent with kin selection.

## 1. Introduction

The predominant social interaction among plants, other than mating, is competition (Kelly 1996) for light, water and nutrients. Vegetative reproduction and self-fertilization can cause groups of plants to be more closely related than groups of animals (Wade 1980), increasing the potential for kin selection. Kin selection theory recognizes that individuals increase their inclusive fitness through behaviour that increases the fitness of related individuals (Hamilton 1964). While Hamilton's rule is usually invoked to explain altruism, it also applies to competition. If kin compete less with each other, individuals increase their direct fitness by not spending resources on competition, and their indirect fitness by not reducing the fitness of neighbouring relatives (Axelrod & Hamilton 1981). Kin selection is facilitated by kin recognition, which allows organisms to favour relatives preferentially over strangers, reducing the costs of positive interactions (Waldman 1988). Kin and other multilevel selection has been demonstrated in plants (Donohue 2003, 2004; Goodnight 1985; Stevens *et al*. 1995; Kelly 1996), and self-incompatibility systems allow plants to discriminate against relatives in mating (Waldman 1988). To our knowledge, however, no studies have yet tested directly for kin recognition in plants.

Plants sense the presence of other plants and respond by producing more competitive phenotypes (Callaway 2002). Phytochrome-mediated stem elongation in response to the red to far-red ratio (R : FR) increases competitive ability in high density (Dudley & Schmitt 1996). However, the lowered R : FR cue resulting from the light absorbance by chlorophyll (Smith 1995) does not convey additional information about the neighbour. Plants increase root allocation in the presence of neighbour roots (Gersani *et al*. 1998, 2001; Maina *et al*. 2002; Falik *et al*. 2003; O'Brien *et al*. 2005; Murphy & Dudley 2007) increasing below-ground competitive ability (Gersani *et al*. 2001). Considerable specificity appears to be conveyed by roots: the growth patterns of roots have shown to depend on neighbour genotype (Mahall & Callaway 1996; Holzapfel & Alpert 2003), neighbour species (Huber-Sannwald *et al*. 1996), whether neighbouring roots are self- or non-self (Mahall & Callaway 1992) or connected by stolons (Holzapfel & Alpert 2003), even in genetically identical individuals (Falik *et al*. 2003; Gruntman & Novoplansky 2004). Here we ask if the root allocation response to the roots of neighbours depends on relatedness.

## 2. Material and methods

*Cakile edentula* var. *lacustris* (Brassicaceae), the Great Lakes sea rocket, is a self-fertilizing annual found along dunes and beaches of the Great Lakes (Rodman 1974). Its fruit structure results in seed dispersal into solitary individual, groups of strangers and groups of siblings (Donohue 2003). Sibling groups have been shown to have higher fitness than groups of strangers in the field (Donohue 2003). On 4 October 2005, collected field-pollinated maternal sibships (hereafter, families) were sampled randomly from one population at Confederation Park in Hamilton, Ontario.

The experimental units were groups of four plants, arranged randomly into six 30×30 cm trays of 64 plants. Density was high (689 plants m^−2^, or 3.81 cm apart), comparable with clumps in the field. In the solitary treatment, the four plants were planted singly in small pots (3.8×3.8×35.56 cm). In the root neighbours treatment, the four plants were planted together in a large pot (7.6×7.6×35.56 cm). The groups were either kin (from the same family) or strangers (from four different families). Each tray had a different subset of four from the eight families used. Families were equally represented in the root and kin treatments within each tray. We used rectangular, open-ended, bleach-board pots (Zipset Plant Bands, Stuewe and Sons, Corvallis) so that density, average resources per plant, soil depth and soil volume were kept constant. Variation in above-ground competition could result in confounding effects of stem elongation on root allocation (Cipollini & Schultz 1999).

On 22 November 2005, the seeds were planted in three parts coarse sand and one part Turface (Profile Products LLC, Buffalo Grove, IL) in a growth room under fluorescent and incandescent lighting. The plants were watered daily and fertilized weekly with 200 ppm 15–15–30 NPK. We harvested early in reproduction at eight weeks, so that root allocation during rapid vegetative growth could be estimated. Plants were partitioned into coarse roots, fine roots, stems, leaves and reproductive tissues (seeds, fruits, and supporting stems and pedicels).

### (a) Statistical analysis

All statistical tests were performed with SAS software, v. 8.02, for Windows. The data were transformed *f*(*x*)=(log(*x*+1)), so that the residual variance was homoscedastic and the distribution of the residuals did not differ significantly from normality. Parameters are presented untransformed for clarity. Because roots in large pots could not be separated, for total mass and root allocation the observation is the group of four (*n*=96). For reproductive allocation, the observation is the individual (*n*=332).

Effects of treatments on biomass allocation were compared for least square means (LSMEANS option of PROC GLM) from analysis of covariance (ANCOVA; Gedroc *et al*. 1996; McConnaughay & Coleman 1999). Kin and stranger means were compared within each root neighbour treatment, which also avoids potential pot-size biases (Hess & De Kroon 2007). For root allocation, fine root mass was the dependent variable, and leaf mass the covariate (table 1). These traits are most appropriate because they function in above-ground and below-ground resource acquisition (Givnish 1986), but root: shoot mass showed the same results. For reproductive allocation, flower mass was the dependent variable and aboveground vegetative mass was the covariate, with a second-order term included.

## 3. Results

Fine root mass was positively associated with leaf mass. The root treatment affected the slope of the relation between fine root mass and leaf mass, and kin treatment affected both the slope and the *y*-intercept (table 1). Comparisons of root allocation (figure 1), showed that kin groups allocated less to their fine root mass than did stranger groups in shared pots (*n*=96, *t*=2.71, *p*<0.0081; figure 1*a*). For those groups in solitary pots, kin and stranger root allocation did not differ (*n*=96, *t*=0.00, *p*<0.9961; figure 1*a*).

Total biomass was greater in root neighbour pots (figure 1*b*; *n*=96, *F*_1,87_=31.7, *p*<0.0001), but was not affected by relatedness (*F*_1,87_=1.26, *p*<0.2649) nor was there a neighbour×relatedness interaction (*F*_1,87_=1.29, *p*<0.2585). Allocation to reproduction responded to the presence of root neighbours (figure 2) but not to relatedness, with the greatest flowering allocation in solitary plants (least square means for individual plants: root neighbours=0.053, solitary plants 0.073; *n*=332, *t*=31.7, *p*<0.0013).

## 4. Discussion

We found that kin groups allocated less to their fine root mass than did stranger groups when they competed below ground, indicating that these plants could discriminate relatives. Root allocation did not differ between kin and stranger groups grown in isolated pots, indicating that the cues for kin recognition lie in root interactions. Siblings were less competitive than strangers, which is consistent with kin selection.

We found plasticity to kin versus strangers only for root competitive ability. Our experimental design did not allow us to assess lifetime fitness, but we did measure total biomass and allocation to reproduction at early reproduction. Neither demonstrated plasticity to kin, though both responded to the presence of neighbours. Previous studies of root allocation responses to neighbours have found that plants in shared pots also had reduced fitness (Gersani *et al*. 2001; Maina *et al*. 2002; O'Brien *et al*. 2005; though see Murphy & Dudley 2007), indicating a cost to increased root allocation. Because sibling groups avoided this potential cost, these results agree with the greater fitness of sibling groups in *C. edentula* found by Donohue (2003). However, we saw no direct trade-off between root allocation and reproduction.

If kin discrimination via root–root interactions proves widespread, it will profoundly change how we view competition in plants. Our results, because we used maternal sibships, indicate a genetic or maternally derived mechanism for kin recognition involving root communication. However, the mechanism is probably different from the self/non-self mechanism, because plants recognize genetically identical individuals as non-self (Falik *et al*. 2003; Gruntman & Novoplansky 2004). Having found kin discrimination once, we expect to find kin discrimination elsewhere in plants, since variable dispersal, variable competitive situations, and increases in fitness when competing with kin, are found in other plants. Other competitive traits, such as stem elongation and apical dominance, are the most probable candidates to exhibit plastic responses contingent on kinship of neighbours.

## Figures and Tables

**Figure 1:**
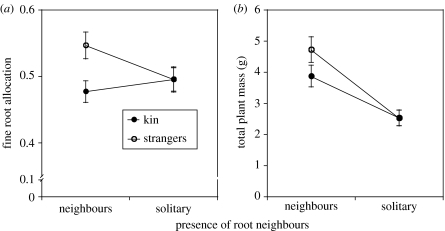
(*a*) Root allocation and (*b*) total mass for groups of four *C. edentula* plants grown either in single pots (solitary) or in one larger shared pot (root neighbours). The groups were either siblings (kin) or from four different maternal families (strangers). Root allocation is the least square mean from an ANCOVA with fine root mass as the dependent variable and leaf mass as the covariate (*n*=96). Bars indicate 1 s.e.

**Figure 2:**
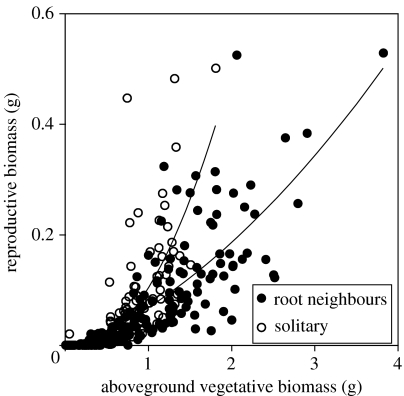
Scatter plot of reproductive mass versus aboveground vegetative mass for single plants of *C. edentula*. Lines indicate second-order regressions of reproductive mass on vegetative mass for each root treatment. No significant kin or kin×root effects were found. *n*=332.

**Table 1:** Analysis of covariance for the derivation of root allocation, with log (fine root mass +1) as the dependent variable, and kin treatment (sibling or stranger), root treatment (neighbours or solitary) and kin×root as main effects. Log (leaf mass +1) was the covariate, and all leaf mass by main effects were estimated. The observation is the biomass of the group of four plants (*n*=96).

source	d.f.	sums of squares	mean square	*F*	*p*
log leaf	1	1.173	1.173	484	0.0000
root	1	0.0076	0.0076	3.13	0.0808
logleaf×root	1	0.0107	0.0107	4.44	0.0381
kin	1	0.021	0.021	8.68	0.0042
logleaf×kin	1	0.014	0.014	5.98	0.0166
root×kin	1	0.000	0.000	0.01	0.9042
logleaf×root×kin	1	0.0005	0.0005	0.21	0.6456
tray	5	0.093	0.0187	7.75	0.0000
error	83	0.200	0.0024		

## Supplementary Material

Supporting statistical tests A brief discussion of methodological and statistical checks for potential bias in the root allocation results
