# Supplementary material for: Kin recognition in an annual plant
Source: Biol Lett. 2007 Jun 13;3(4):435–8. doi: 10.1098/rsbl.2007.0232 (PMC2104794; doi:10.1098/rsbl.2007.0232)
Supplement: Supplementary file 1 [file rsbl20070232s20.doc]

Plants were labeled by location, so no one was aware during processing or biomass weighing of the family or kin treatment.

Family effects are not estimated in this analysis. Instead, for each tray, the experimental design balanced representation of the four families within the four treatment combinations. Because there were individuals that died, and success of replacement seeds was low, imbalance among the genotypes for the different treatment combinations is a potential confounding factor. Several analyses were done to test for this possibility. We found by inspection and, more formally, through a chi-square analysis that the genotypes were not biased in their distribution among the root×kin treatments. Within the root neighbours treatments, groups with 2 or 3 plants did not differ in mass from groups of four. We dropped groups with two or three missing plants and found similar significance levels. We tested for genetic differences among families in root allocation by examining the solitary pots and the root neighbour pots with siblings only, and found no among-family difference. These among-family effects were necessarily nested within trays, because each tray contained a unique combination of families. We examined the data for outliers. No one tray was an outlier for high levels of root allocation. One tray was unusual in that sibling and stranger groups showed the same low levels of root allocation; when this tray is dropped from the analysis, the significance levels for the difference between lsmeans of kin and strangers in root neighbours was increased.
